# Longitudinal Trajectories of Memory Performance in Patients with Early-Stage Breast Cancer

**DOI:** 10.1155/2022/5899728

**Published:** 2022-04-16

**Authors:** Alexandra C. Apple, Cutter A. Lindbergh, Susan M. Landau, Amy DeLuca, Jamie L. Eberling, William J. Jagust, Joel H. Kramer, Hope S. Rugo, Lara H. Heflin

**Affiliations:** ^1^University of California San Francisco, Department of Neurology, Memory and Aging Center, San Francisco, CA, USA; ^2^Department of Psychiatry, Connecticut School of Medicine, USA; ^3^School of Public Health and Helen Wills Neuroscience Institute, University of California Berkeley, Berkeley, CA, USA; ^4^Molecular Biophysics and Integrated Bioimaging, Lawrence Berkeley National Laboratory, Berkeley, CA, USA; ^5^University of California San Francisco Helen Diller Family Comprehensive Cancer Center, San Francisco, CA, USA; ^6^PhD Michael J. Fox Foundation New York, NY, USA; ^7^New Mexico Highlands University, Department of Psychology, Las Vegas, NM, USA

## Abstract

**Background:**

While breast cancer and its treatments may affect cognition, the longitudinal trajectories of cognition among those receiving differing cancer treatment types remain poorly understood. Prior research suggests hippocampal-prefrontal cortex network integrity may influence cognition, although how this network predicts performance over time remains unclear.

**Methods:**

We conducted a prospective trial including 69 patients with early-stage breast cancer receiving adjuvant therapy and 12 controls. Longitudinal cognitive testing was conducted at four visits: pretreatment-baseline, 6-7 months, 14-15 months, and 23-24 months. Cognitive composite scores of episodic memory, executive functioning, and processing speed were assessed at each timepoint. Baseline structural MRI was obtained in a subset of these participants, and hippocampal and prefrontal cortex regional volumes were extracted.

**Results:**

Longitudinal linear mixed modeling revealed significant group by time interactions on memory performance, controlling for age and education. Post hoc analyses revealed this effect was driven by patients treated with chemotherapy or chemotherapy plus hormone therapy, who demonstrated the least improvement in memory scores over time. Treatment group did not significantly influence the relationship between time and processing speed or executive functioning. Neither pretreatment hippocampal nor prefrontal volume differed between groups, and there were no significant group by time by baseline regional volume effects on cognition.

**Conclusion:**

Patients with early-stage breast cancer treated with chemotherapy or chemotherapy plus hormone therapy benefit less from practice effects seen in healthy controls on memory tests. Loss of longitudinal practice effect may be a new and clinically relevant measure for capturing patients' experience of cognitive difficulties after treatment.

## 1. Introduction

Approximately 75% of individuals undergoing treatment for cancer report cognitive impairment, with up to 35% of cancer survivors experiencing cognitive impairment for months to years following the completion of treatment [1, 2]. This decline in cognitive ability, termed Cancer-Related Cognitive Dysfunction (CRCD), cannot be solely attributed to depression, stress, or fatigue [3–5]. Cognitive features of CRCD can include difficulties in several cognitive domains, including memory, executive function, attention, and processing speed [6–9]. A few studies have sought to characterize longitudinal cognitive trajectories and have found conflicting evidence of longitudinal declines and improvements over time in aspects of cognition [10, 11]. There is a need for additional research on longitudinal trajectories of objective cognitive domains before, during, and after treatment. A clearer understanding of changes in cognition will help untangle the differential impact of cancer treatments on brain functions [12].

Many studies show cross-sectional differences between cancer patients and controls in structural MRI during or after cancer treatment [11]. The hippocampus and prefrontal cortex (PFC) are known to be integrally involved in memory and executive functioning and are particularly sensitive to the effects of cancer treatments including chemotherapeutic agents [13–18]. Specifically, research has shown longitudinal reductions in hippocampal volumes and abnormalities in gray and white matter following breast cancer diagnosis and treatment [16]. In dementia literature as well as healthy aging literature, larger brain structure has been shown to confer protection from cognitive decline [19]. However, little is known about how brain structure prior to the start of cancer treatment may affect cognitive trajectories.

This study is aimed at evaluating the acute and relatively long-term effects (about 2 years) of chemotherapy and/or hormonal therapy on cognitive function in women with early-stage breast cancer. Specifically, this study is aimed at exploring whether specific cognitive domains (memory, processing speed, and executive function) differ over time between controls and breast cancer patients treated with chemotherapy and hormone therapy, hormone therapy alone, and chemotherapy alone. Breast cancer, the most common malignancy in women worldwide, is an ideal disease for this study as most patients receive adjuvant therapy and are long-term survivors of this disease. We hypothesized that patients with breast cancer would demonstrate poorer performances in memory, processing speed, and executive function, over time, compared to controls. Secondly, we sought to assess whether hippocampal and PFC volumes predict differing trajectories of change in cognition over time. We hypothesized that patients with breast cancer with larger relative brain structure would be less vulnerable to treatment-related deficits in cognitive performances over time.

## 2. Methods

### 2.1. Participants

Participants were recruited through the Breast Care Center at the University of California San Francisco (UCSF) Comprehensive Cancer Center. Eligibility included ages 35-80, a diagnosis of stage I, II, or III breast cancer where treatment was recommended in one of 3 cohorts: chemotherapy and hormone therapy (CT+HT), hormone therapy alone (HT), or chemotherapy alone (CT). Hormone therapy could include an aromatase inhibitor (anastrozole, exemestane, or letrozole) or tamoxifen. Eligible chemotherapy regimens had a duration of 3 to 4 months and included anthracycline and taxane with or without trastuzumab; docetaxel and cyclophosphamide with or without trastuzumab; doxorubicin and cyclophosphamide; or docetaxel, carboplatin, and trastuzumab. Patients under the age of 35 were excluded due to the marked variability in disease characteristics, genetics, and prognosis in very young women diagnosed with breast cancer and to limit to some degree the heterogeneity of the studied population. Controls were recruited from the community using advertisements in the hospital and local newspapers. Eligibility criteria for controls included female gender, ages 35–80 years old, no history of cancer treatment or major medical or psychologic illness, and no prescription medication use. Exclusion criteria for all groups included prior treatment with chemotherapy, brain radiation, or intrathecal therapy, history of major psychiatric illness, head injury, neurological disorder (e.g., epilepsy, large vessel stroke, and multiple sclerosis), or drug or alcohol use disorder.

### 2.2. Procedure

After obtaining informed consent, participants underwent neuroimaging and neuropsychological evaluations and answered a variety of self-report questionnaires. Neuropsychological assessments were conducted at four timepoints: prior to the start of adjuvant treatment (pretreatment baseline, T1), after beginning treatment at approximately 6 months (T2), 15 months (T3), and 2 years (T4) after the baseline assessment. A 6-month time frame for T2 was based on key factors in the treatment of breast cancer, primarily extensive short-term use of medications that impact cognition to manage the toxicity of acute therapy, and the length of time on adjuvant endocrine therapy. In addition, our goal was to capture longer-term effects that do not resolve after completing chemotherapy. The neuropsychological assessment included self-report measures of depression, anxiety, fatigue, and perceived cognitive functioning, as well as performance-based neuropsychological tests (described in detail below). Demographic characteristics were collected at baseline, including age, education, ethnicity, and marital status. Bioavailable estradiol was collected at T1, T2, and T4. A subset of patients and controls underwent neuroimaging to evaluate structural changes in the brain. Participants provided written informed consent, and this study was approved by UCSF's Committee on Human Research.

### 2.3. Neuropsychological Measures

Participants were administered a neuropsychological battery that included the following tests: California Verbal Learning Test, 2^nd^ Edition (CVLT-II; Delis, Kramer, Kaplan, & Ober, 2000); Wechsler Adult Intelligence Test, 3^rd^ Edition (WAIS-III; Wechsler, 1997) Digit Symbol; Delis-Kaplan Executive Function System (D-KEFS; Delis, Kaplan, & Kramer, 2001) Verbal Fluency Test, Trail Making Test, and Color-Word Interference Test; and a computerized Continuous Performance Test (CPT). These tests are similar to those recommended by the International Cognition and Cancer Task Force (ICCTF), although this study began data collection before publication of ICCTF recommendations [20]. Raw test scores were transformed to *z*-scores based on baseline timepoints. Items within each of the composites were reverse scored if necessary, so that a higher score indicated better performance. Tests were combined into composite scores in an effort to reduce measurement error which may accompany analyzing individual tests. Composite scores were modeled after validated composites which have been used in longitudinal studies [21].

The processing speed domain consisted of CPT median reaction time and D-KEFS Color-Word Interference Test time (trials 1 and 2), WAIS-III Digit Symbol, and Trail Making Test condition 5 time. The memory composite was made up of several scores from the CVLT-II including short delay free recall, long delay free recall, trials 1-5, recognition hits, and recognition false positives. Finally, the executive functioning domain consisted of Verbal Fluency Test (FAS), D-KEFS Color-Word Interference (trials 3 and 4), and Trail Making Test 4.

### 2.4. MRI

Due to changes in scanners and extensive missing data, only baseline MRI was used in the current analysis. A subset of participants underwent structural MR imaging at baseline on a Siemens 1.5T TIM TRIO scanner with an 8-channel dedicated head coil at UCSF or a Siemens 1.5T AVANTO scanner with a 12-channel dedicated head coil at Lawrence Berkeley National Laboratory (C + H = 13, HT = 13, CT = 8, and controls = 10). Structural imaging was acquired using T1-weighted scans (UCSF: Fast SPGR, TR = 7 ms, TE = 3.01 ms, voxel size = 1 mm^3^, FOV = 24 cm, flip angle = 25°, and 160 axial slices and UC Berkeley: TR = 2110 ms, TE = 3.58 ms, voxel size = 1 mm^3^, FOV = 25.6 cm, flip angle = 15°, and 160 axial slices). Participants' structural images were processed with the atlas-based Free Surfer (version 5.3) to produce hippocampal volumes, as well as prefrontal lobe volume.

The Desikan-Killiany atlas [22] was used to create 35 cortical regions of interest (ROIs) in each of the hemispheres. Left and right hippocampal ROI was extracted from this atlas and combined to create a bilateral hippocampal ROI for each participant. For prefrontal cortical analyses, 8 bilateral regions were identified as part of the prefrontal cortex; these include the superior frontal, caudal middle frontal, rostral middle frontal, lateral orbitofrontal, frontal pole, rostral anterior cingulate, caudal anterior cingulate, and medial orbitofrontal [22, 23]. Total intracranial volume was extracted and controlled for in the analyses.

### 2.5. Statistics

We aimed to recruit 30 C+H patients, 30 HT patients, 20 CT patients, and 20 age-matched healthy control women. Baseline group differences on demographic and self-reported characteristics were tested with chi-square tests (*χ* statistics) and analysis of variance (ANOVA, *F* statistics) where appropriate. To address the primary aim of this study, longitudinal linear mixed modeling was used to assess trajectories of change in cognitive domains over time by group, after controlling for baseline covariates. This statistical analysis approach is consistent with ICCTF recommendations for analyzing longitudinal data [20]. Longitudinal linear mixed modeling was also used to assess for group by time by baseline hippocampal or frontal lobe volume interactions on cognition after controlling for intracranial volume, MRI site, and baseline covariates. Statistical computations were performed using Stata 15.1 (StataCorp, College Station, TX, USA).

## 3. Results

### 3.1. Demographics and Self-Reported Mood

**Figure 1:**
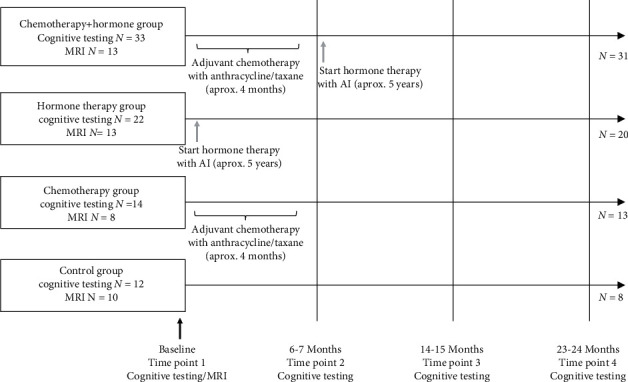
Timeline of study timepoints relative to treatments in cancer and control groups. AI: aromatase inhibitor (anastrozole, exemestane, or letrozole).

Patient demographic data is represented in Table 1. Cancer-related data, including treatment regimens, are represented in Table 2. The number of patients with neuropsychological testing and MRI testing at each timepoint is outlined in Figure 1. A total of 69 patients with early breast cancer and 12 controls were included at baseline. Patients were enrolled in one of three groups: 33 patients underwent both chemotherapy and hormone therapy (C+H), 22 underwent hormone therapy alone (HT), and 14 underwent chemotherapy alone (CT). By T4, the C+H and HT groups each had two participants drop out, the CT group had one participant drop out, and the control group lost four participants.

There were nonsignificant but subtle differences in baseline age between the groups (*F*(df) = 2.72 (3), *p* = 0.05), with the HT cohort demonstrating older age than the controls. There were no significant differences in education, ethnicity, or marital status ((*F*(df) = 1.20 (3), *p* = 0.31); *χ*^2^(df) = 16.59 (12), *p* = 0.17; *χ*^2^ (df) = 11.59 (9), *p* = 0.24). HR expression and HER2 expression, estradiol level, menopausal status, and treatment regimens are outlined in Table 2. Forty-five percent of the C+H group, 63% of the HT group, and 71% of the CT group were premenopausal at the start of the study. Participant's estrogen (serum estradiol pg/mL) levels are reported in Table 2 as well (*N* = 70). Chemotherapy regimens consisted of anthracycline+taxane doxorubicin/cyclophosphamide followed by paclitaxel±trastuzumab, paclitaxel/carboplatin followed by doxorubicin/cyclophosphamide±trastuzumab, paclitaxel/neratinib followed by doxorubicin/cyclophosphamide±trastuzumab, or paclitaxel followed by doxorubicin/cyclophosphamide), cyclophosphamide+paclitaxel (paclitaxel/carboplatin±trastuzumab or paclitaxel/trastuzumab), or other (gemcitabine/carboplatin followed by paclitaxel/carboplatin). Hormone therapy regimens consisted of aromatase inhibition (anastrozole, letrozole, or exemestane) or tamoxifen or both. Three of the participants in the C+H group and 4 in the HT group received ovarian suppression.

In terms of self-reported mood symptoms at baseline (Table 3), there were significant group differences in depression between groups (*F*(df) = 2.99 (3), *p* = 0.04), with the patient groups demonstrating significantly more depression compared to the control group although scores were within the normal range. There was no significant difference in anxiety (state: *F*(df) = 1.07 (3), *p* = 0.37; trait: *F*(df) = 1.12 (3), *p* = 0.35) or baseline fatigue (*F*(df) = 1.42 (3), *p* = 0.24) between groups.

### 3.2. Cognitive Performance

We controlled for age, education, and depression given the known effects that these variables often have on cognitive performance, as well as the statistically greater amount of depression observed in cancer groups in the currently analysis. Pretreatment baseline and longitudinal cognitive *z*-scores are represented in Supplementary Table 1. At baseline, there was a significant group difference in memory performance between the patient and the control groups (*F*(df) = 3.19 (4), *p* = 0.02). Post hoc analysis, which examined the three cancer groups individually, revealed that this effect was driven by group differences between controls and CT as well as controls and C+H, with controls having worse baseline memory. There were no significant group differences in cognitive performance in executive function or processing speed.

**Figure 2:**
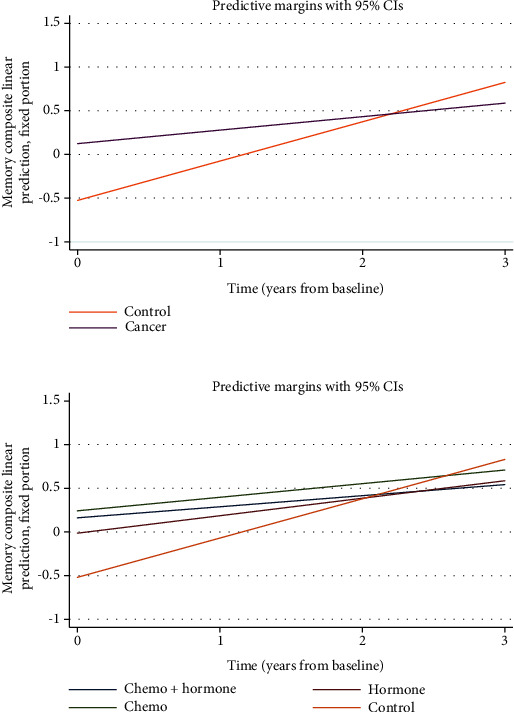
Interaction between time and group on memory performance. Group membership ((a) cancer vs. controls and (b) Chemo+hormone, hormone, chemotherapy vs. controls) attenuates the relationship between time and memory performance such that controls demonstrated more improvement in memory over time compared to cancer patients. Model-predicted memory composite scores are represented by sample-based *z*-scores.

In terms of longitudinal analyses, we found significant main effects of group (*β* = 0.50, *z* = 2.74, and *p* = 0.006) and time (*β* = 0.47, *z* = 4.05, and *p* < 0.001) on memory performance. There was a statistically significant group by time interaction on memory performance (*β* = −0.31, *z* = −2.35, and *p* = 0.013; Figure 2(a)) such that controls demonstrated better performances over time compared with the patients. Post hoc analysis revealed that this effect was driven by patients who had received chemotherapy, i.e., the CT and C+H groups combined (*β* = −0.33, *z* = −2.28, and *p* = 0.022), with those with the control group demonstrating improvements in memory over time compared with CT and C+H (Figure 2(b); all treatment groups are represented for illustrative purposes). We did not observe significant group by time interactions in processing speed or executive function domains.

Because controls demonstrated lower memory scores at pretreatment baseline, a sensitivity analysis was conducted which matched baseline memory scores (C+H *n* = 29, HT *n* = 18, CT *n* = 11, and control *n* = 12). Even in this smaller cohort significant main effects of group (*β* = 0.33, *z* = 2.07, and *p* = 0.040), time (*β* = 0.47, *z* = 3.90, and *p* < 0.001) as well as the interaction of group by time on memory remained (*β* = −0.28, *z* = −2.08, and *p* = 0.038).

### 3.3. MRI

Due to changes in scanners (two different scanners were used and data could not be harmonized) and imaging parameters longitudinally which introduced noise that could not be statistically corrected, only baseline data was used in a subset of participants. This subset included 34 breast cancer patients and 10 controls. There were no group differences between groups on hippocampal volume or PFC volume at baseline after controlling for baseline age, education, depression intracranial volume, and MRI site difference at baseline. In terms of longitudinal analyses, there were no significant groups by time by baseline hippocampal or frontal lobe volume interactions on memory, processing speed, or executive functioning performance.

## 4. Discussion

This study examined the longitudinal effects of CRCD by characterizing differences in cognitive trajectories in patients with early-stage breast cancer receiving standard adjuvant therapy compared with controls. Controls demonstrated improvements in memory performance over time, whereas those who underwent cancer treatment did not exhibit the same degree of improvement. Specifically, the patients receiving chemotherapy, either with or without hormone therapy, appeared to have the greatest reduced efficiency in learning over time, with flatter trajectories compared to controls. We also examined the effects of pretreatment hippocampal volumes as well as prefrontal lobe volumes on trajectories of cognitive performance but did not find significant group differences. Similarly, pretreatment hippocampal and PFC volumes did not differ across groups, adding to the mostly consistent published findings that cancer patients do not demonstrate structural MRI differences prior to treatment [24].

The question of whether cognitive changes occur before and after treatment for early-stage breast cancer has been a subject under scrutiny for several decades [11]. A recent review of the literature found that breast cancer survivors tested 1-4 years postchemotherapy generally show within-group improvements in some domains including verbal memory and processing speed [11, 25, 26]. Our findings add to this body of work in that they demonstrate subtle effects of cancer treatment on memory trajectories. While healthy controls continue to show improved results with repeated cognitive testing (i.e., practice effects), patients with early-stage breast cancer receiving adjuvant therapy demonstrate a much flatter performance slope over time. These results are similar in nature with others which have found declining cognitive performances in cancer patients compared to controls [27, 28]. Unlike several studies which show subsets of patients with breast cancer performing worse on cognitive tests prior to starting adjuvant treatment [24, 29, 30], the cancer group in the current analysis demonstrated relatively better memory performance compared with controls at a pretreatment baseline. Practice effects, although historically viewed as a source of measurement error, have more recently been shown to demonstrate prognostic value in clinical groups [31]. The evidence that the patients with breast cancer in our study appear to benefit less from practice effects compared to controls lends support to the subjective reports of cognitive difficulties that many breast cancer patients endorse during and after treatment and, although subtle, point to objective changes in memory performance.

Why might this observed lack of practice effect be more specific to patients receiving chemotherapy? Although the mechanisms are unclear, studies have found that chemotherapy agents may be particularly harmful to memory performance and memory networks. Chemotherapy is thought to amplify amyloid beta plaque accumulation by altering glucose metabolism and causing cytokine-mediated inflammation, oxidative stress, and blood vessel damage [32–34]. Animal studies also suggest a link between chemotherapy and cognition [33, 35]. One study by Seigers and colleagues found chemotherapy-induced cellular damage in the hippocampus and behavioral impairment in visuospatial memory in mice treated with methotrexate [33]. In future studies, it may be useful to examine whether diminished practice effects in patients treated with chemotherapy for early-stage breast cancer correlate with longitudinal imaging changes in hippocampal or other memory-related regions.

Studies examining brain changes in the aging population have posited that individuals with more capacity (e.g., thicker cortex and less atrophy) are buffered against the effects of age-related cognitive decline [36, 37]. We hypothesized that the same effects would be seen in patients with early-stage breast cancer such that larger pretreatment baseline hippocampal and PFC volumes would be linked to practice effects comparable to those seen in controls. Contrary to our hypothesis, we did not find differences in cognitive trajectories based on baseline volumes. Even patients with larger frontal lobes and hippocampus did not reveal the practice effects that were seen in our control patients. Given the small number of subjects who underwent imaging as a part of the current study and the lack of longitudinal imaging, this finding is limited in power to detect small associated differences. Although no differences were observed in pretreatment baseline hippocampal or PFC volumes between groups, it is possible that other changes including aberrant activation and functional or structural connectivity may be present in breast cancer survivors. A study by Ryals and colleagues found deficits in overt and covert spatial familiarity-based recognition memory relating to decreased hippocampal activity in premenopausal breast cancer patients treated with chemotherapy and tamoxifen [38]. Another study in the same cohort of patients found increased connectivity between the hippocampus and precuneus to be related to subjective concern in patients with breast cancer [39]. More imaging research utilizing multiple modalities over time is necessary to better understand the impact CRCD has on the brain and vice versa.

A limitation of the current study is the small sample size of both the treatment and control groups and the fact that MR imaging was only evaluable in a subset of participants. The smaller sample size also did not allow for evaluating differential effects from specific chemotherapy regimens. Unfortunately, it was not feasible to follow the ICCTF recommendations for specific neuropsychological test as these recommendations were made after the current study began, although many of the included tests are quite similar in nature to those recommended by the ICCTF (e.g., HVLT and CVLT). Another caveat to this study is the observation that pretreatment baseline memory scores were lower in the control group compared to the cancer group, although this finding is limited by the small number of control patients. The sensitivity analysis in a subset of participants for whom there was no difference in baseline memory helped to assure that this effect was not purely a regression to the mean. Nevertheless, future studies would benefit from recruiting participants who are matched for baseline cognitive performance, although this would be extremely challenging. Finally, as stated above, future studies should explore longitudinal markers of other brain mechanisms that may be a target in CRCD, including aspects of functional MRI including connectivity and activation.

In summary, the primary finding of our study is that, when compared to patients with early-stage breast cancer receiving adjuvant therapy, healthy controls demonstrated significantly better memory trajectories over time. Post hoc analyses revealed that this effect was driven by patients who received chemotherapy either with or without subsequent hormone therapy. We posit that this lack of improvement may be experienced by patients as subtle disadvantages in memory performance over time, particularly for those treated with chemotherapy. This lends support to the subjective cognitive concerns that many patients report during and after treatment. Given the variability in studies of neuropsychological deficits in patients with breast cancer, loss of longitudinal practice effect may be a new measure for capturing the experience of cognitive difficulties during and after treatment for breast cancer.

## Figures and Tables

**Table 1:** Patient demographics at pretreatment baseline.

	Chemotherapy+hormone therapy group *N* = 33	Hormone therapy group *N* = 22	Chemotherapy group *N* = 14	Control group *N* = 12	ANOVA *F*/*χ*^2^ (df)	*p* value
Demographics Mean (SD)						
Age	53.61 (7.43)	56.35 (7.19)	51.64 (7.82)	48.75 (9.68)	2.72 (3)	0.05
Years of education	17.09 (1.88)	16.55 (2.24)	16.71 (2.43)	15.67 (2.93)	1.20 (3)	0.31
Ethnicity (*N*)					16.59 (12)	0.17
White	29	19	8	7		
Asian/Pacific Islander	2	3	4	3		
Black	1	0	2	2		
Other	1	0	0	0		
Marital status (*N*)					11.59 (9)	0.24
Married	24	18	8	5		
Single	6	2	4	4		
Divorce	3	2	2	2		
Domestic partner	0	0	0	1		

Participant demographics at baseline. *N*: number of subjects; SD: standard deviation; df: degrees of freedom.

**Table 2:** Cancer-related demographics and treatment at baseline.

	Chemotherapy+hormone therapy group *N* = 33	Hormone therapy group *N* = 22	Chemotherapy group *N* = 14	Control group *N* = 12
Menopausal status *N* (percentage of group)				
Premenopausal	15 (45%)	14 (64%)	10 (71%)	--
Postmenopausal	18 (55%)	8 (36%)	4 (29%)	--
Serum estradiol (Median (range))	15 (2-274) *N* = 29	19 (2-132) *N* = 17	14 (2-286) *N* = 13	14 (2-130) *N* = 11
Expression of ER and/or PR *N*				
Positive	33	22	0	--
Negative	0	0	14	--
HER2 *N* (percentage of group)				
Positive	4 (12%)	0 (0%)	3 (21%)	--
Negative	29 (88%)	21 (95%)^∗^	11 (79%)	--
Treatment *N* (percentage of group)				
Chemotherapy				
Anthracycline+taxane	19 (58%)	--	9 (64%)	--
Paclitaxel±cyclophosphamide	12 (36%)	--	3 (21%)	--
Other	2 (6%)	--	2 (14%)	--
Hormone therapy				
Aromatase inhibitor	18 (54%)	15 (68%)	--	--
Tamoxifen	14 (42%)	3 (13%)	--	--
Aromatase inhibitor+tamoxifen	1 (3%)	4 (18%)	--	--

Cancer treatment-related demographics at baseline. ER: estrogen receptor; PR: progesterone receptor; *N*: number of subjects; df: degrees of freedom. Chemotherapy regimens consisted of anthracycline+taxane (doxorubicin/cyclophosphamide followed by paclitaxel±trastuzumab, paclitaxel/carboplatin followed by doxorubicin/cyclophosphamide±trastuzumab, paclitaxel/neratinib followed by doxorubicin/cyclophosphamide±trastuzumab, or paclitaxel followed by doxorubicin/cyclophosphamide), cyclophosphamide+paclitaxel (paclitaxel/carboplatin±trastuzumab or paclitaxel/trastuzumab), or other (gemcitabine/carboplatin followed by paclitaxel/carboplatin). Hormone therapy regimens consisted of aromatase inhibition (anastrozole, letrozole, or exemestane) or tamoxifen or both. ^∗^One participant was missing HER2 data.

**Table 3:** Participant self-reported mood at baseline. Depression was assessed with the Hamilton Depression Rating Scale (HAMD), anxiety was assessed by the State-Trait Anxiety Inventory (STAI), and fatigue was assessed with the Fatigue Symptom Inventory (FSI). In all scales, higher scores represent higher levels of the symptom (e.g., higher levels of depression, anxiety, and fatigue). SD: standard deviation; df: degrees of freedom; ^∗^statistically differs between groups.

Self-report Mean (SD)	Chemotherapy+hormone group *N* = 33	Hormone group *N* = 22	Chemotherapy group *N* = 14	Control group *N* = 12	ANOVA *F* (df)	*p* value
Depression (HAMD)	6.57 (3.30)	5.99 (4.03)	6.09 (3.09)	3.09 (3.38)	2.99 (3)	0.04^∗^
Anxiety (STAI-state)	32.58 (11.22)	31.50 (10.31)	32.14 (6.98)	26.54 (7.17)	1.07 (3)	0.37
Anxiety (STAI-trait)	31.72 (9.38)	34.33 (9.29)	30.93 (6.06)	28.67 (9.28)	1.12 (3)	0.35
Fatigue (FSI total)	35.97 (26.47)	38.82 (24.87)	31.57 (18.79)	21.50 (25.13)	1.42 (3)	0.24

## Data Availability

The data that support the findings of this study are available from the corresponding author upon reasonable request.

## Supplementary Materials

Supplementary Materials Supplemental Table 1: longitudinal cognition composite scores by group. Baseline sample-based *z*-score group differences are shown, as well as *z*-scores for timepoints 2-4.
