# Supplementary material for: Longitudinal Trajectories of Memory Performance in Patients with Early-Stage Breast Cancer
Source: J Oncol. 2022 Apr 16;2022:5899728. doi: 10.1155/2022/5899728 (PMC9034940; doi:10.1155/2022/5899728)
Supplement: Supplementary Materials — Supplemental Table 1: longitudinal cognition composite scores by group. Baseline sample-based z-score group differences are shown, as well as z-scores for timepoints 2-4. [file 5899728.f1.docx]

| Cognition Composite  z score Mean (SD) | Chemotherapy + Hormone | Hormone Group | Chemotherapy Group | Control Group | ANOVA F (df) Cancer vs Control | p value |
| --- | --- | --- | --- | --- | --- | --- |
| Processing Speed |  |  |  |  |  |  |
| Baseline | 0.007 (0.52) | -0.180 (0.73) | 0.161 (0.60) | 0.078 (0.65) | 1.57 (4) | 0.19 |
| T2 | -0.042 (0.70) | -0.005 (0.71) | 0.399 (0.89) | 0.201 (0.48) |  |  |
| T3 | -0.049 (0.77) | 0.048 (0.66) | 0.618 (0.40) | -0.108 (0.48) |  |  |
| T4 | 0.009 (0.89) | -0.109 (0.91) | 0.556 (0.57) | 0.144 (0.53) |  |  |
| Memory |  |  |  |  |  |  |
| Baseline | 0.070 (.65) | -0.002 (0.86) | 0.269 (0.75) | -0.501 (0.86) | 3.19 (4) | 0.02* |
| T2 | 0.324 (0.63) | -0.092 (0.82) | 0.370 (0.63) | -0.202 (0.97) |  |  |
| T3 | 0.384 (0.66) | 0.278 (0.82) | 0.536 (0.84) | 0.147 (0.73) |  |  |
| T4 | 0.416 (0.82) | 0.335 (0.56) | 0.559 (0.42) | 0.487 (0.59) |  |  |
| Executive Function |  |  |  |  |  |  |
| Baseline | 0.247 (.54) | -0. 228 (0.89) | 0.110 (0.46) | -0.247 (0.96) | 1.11 (4) | 0.36 |
| T2 | 0.061 (0.80) | 0.021 (0.74) | 0.334 (0.29) | 0.107 (0.47) |  |  |
| T3 | 0.081 (0.74) | 0.178 (0.61) | 0.597 (0.36) | -0.020 (0.71) |  |  |
| T4 | 0.149 (0.97) | 0.027 (0.81) | 0.238 (0.96) | -0.202 (0.80) |  |  |

Supplemental Table 1. Longitudinal cognition composite scores by group. Baseline sample-based z-scores
